# Improvement of the quality of *BRAF* testing in melanomas with nationwide external quality assessment, for the BRAF EQA group

**DOI:** 10.1186/1471-2407-13-472

**Published:** 2013-10-11

**Authors:** Jean-François Emile, Julie Tisserand, Loic Bergougnoux, Frédérique Nowak, Gladwys Faucher, Sylvie Surel, Aude Lamy, Delphine Lecorre, Zofia Helias-Rodzewicz, Paul Hofman, Jean-Christophe Sabourin, Pierre Laurent-Puig

**Affiliations:** 1 EA4340, Versailles SQY University, Boulogne, France; 2 Department of Pathology, Ambroise Pare Hospital, APHP, 9 Av. Charles de Gaulle, Boulogne F-92104, France; 3 Roche, Boulogne, France; 4 Institut National du Cancer (INCa), Boulogne, France; 5 CHU de Rouen, Rouen, France; 6 UMR775, INSERM and Université Paris-Descartes, Paris, France; 7 Laboratory of Clinical and Experimental Pathology, Pasteur Hospital and Medical School, Nice Sophia University, Nice 06002, France

## Abstract

**Background:**

Knowledge about tumour gene mutation status is essential for the treatment of increasing numbers of cancer patients, and testing quality has a major impact on treatment response and cost. In 2012, 4,629 tests for *BRAF* p.V600 were performed in France, in patients with melanomas.

**Methods:**

Two batches of unstained melanoma sections were sent, in May and November 2012, to the 46 laboratories supported by the French National Institute of Cancer (INCa). An external quality assessment (EQA) evaluated mutation status, response times and compliance with INCa recommendations.

**Results:**

All the French laboratories involved in testing participated in the EQA. Fourteen different methods were used to detect *BRAF* mutations, most consisting of combinations of in-house techniques. False responses were noted in 25/520 cases (4.8%), 11 of which concerned confusion between p.V600E and p.V600K. Thus, 2.7% of responses would have led to inappropriate treatment. Within six months, mean response times decreased from 22 to 12 days (*P*<0.001), and the percentage of samples evaluated by a pathologist for tumour cell content increased, from 75.2% to 96.9% (*P*<0.001).

**Conclusion:**

Despite the use of non-certified methods, the false response rate was low. Nationwide EQA can improve the quality of molecular pathology tests on tumours.

## Background

Molecular pathology tests on tumours are increasingly required by clinicians seeking targeted treatments for patients with cancers. The list of targeted therapies is rapidly expanding, and molecular tests are already mandatory to guide treatment decisions for patients with metastatic colorectal carcinomas with EGFR antibodies [1,2], lung carcinomas with EGFR inhibitors [3] and metastatic melanomas with BRAF inhibitors [4]. These tests are usually performed on DNA extracted from formalin-fixed paraffin-embedded (FFPE) tumour samples. In 2005, trastuzumab was shown to improve the survival of patients with breast carcinomas [5,6], and the evaluation of HER2 status became mandatory in patients with such tumours. However, in 2006, central testing for 2535 patients confirmed only 86% of the results obtained in local laboratories [7], and there was still some debate about the reliability of testing for HER2 amplification in 2011 [8]. Thus, since 2005, thousands of patients may have received inappropriate treatment due to false results in mutation tests. The history of *in situ* testing for HER2 should encourage physicians and health authorities to pay attention to the quality of mutation testing in cancers.

Disease-free and overall survival is improved by treatment with the specific inhibitor vermurafenib, in patients with advanced or metastatic melanomas with *BRAF* mutations [4,9]. This has led US and European authorities to approve its use for patients with *p.V600E* or *p.V600* mutations, respectively. Clinical benefit has also been demonstrated for other BRAF and/or MEK inhibitors [10,11], and clinical assays are currently underway in patients at earlier stages of disease. Thus, since June 2011the determination of *BRAF* mutational status has been obligatory, to determine the best treatment options for patients with late-stage melanomas.

In 2008, the French National Institute for Cancer (INCa) initiated a nationwide program for the development of regional platforms for molecular pathology testing for cancers [12]. This national network consists of 28 platforms involving 46 laboratories. These laboratories are supported by grants from INCa, and all the tests are performed free of charge. In 2011, 20,761 *EGFR* tests for lung non-small cell carcinomas and 17,153 *KRAS* tests for colorectal carcinomas (http://www.e-cancer.fr) were performed. In practical terms, any of the 63,703,191 inhabitants of France (http://www.insee.fr) can benefit from molecular pathology analysis for free, provided it is necessary for treatment, and this nationwide program can thus be considered a success. The French platforms performed 3,479 *BRAF* tests on melanoma samples in 2011 and 4,629 in 2012.

This study is the first evaluating the quality of the tests performed in this national network. We used three parameters to assess quality: *BRAF* mutation status, response times and conformity to the French recommendations for test reporting.

## Methods

The EQA involved two independent tests, performed in May and November 2012. The protocol was identical for both tests (May and November): 10 formalin-fixed paraffin-embedded (FFPE) samples with massive lymph node metastases were obtained from the Ambroise Paré Centre for Biological Resources (Boulogne, France). The study was approved by the Ile de France 8 institutional review board (#12 01 08). The samples studied were taken from patients who had already died before the start of the study. Four patients had a p.V600E, one had a p.V600K and five had no *BRAF* p.V600 mutations. The reference mutational status was checked by Cobas [4] and pyrosequencing [13,14]. A dedicated computer program was developed (Lincoln, Boulogne, France) to anonymise each sample and each participating laboratory through the assignment of random code numbers. Each sample received three different identification numbers, to prevent the exchange of information between participating laboratories. For each test, six of the ten samples were selected at random, such that the final set of six samples contained at least two cases with and two cases without *BRAF* mutations. The participating laboratories, EQA organisers and sponsor were unaware of the samples chosen until the databases of collected responses were blocked.

Each of the ten selected FFPE samples was re-embedded in two twin blocks, and the six selected cases were then re-embedded again by an external pathology laboratory (IPP, Paris, France) for anonymisation. Serial 4 μm-thick sections were cut from the same FFPE block where possible, or from the twin block if the first block had already been used up. Sections were cut in a dedicated molecular pathology environment (specific room, equipment, reagents and trained technicians). Slides were received by the participating laboratories within four weeks of cutting. The participating laboratories were aware of the month, but not of the day of testing.

Participation in this external quality control (EQA) study was free of charge. The protocol was sent to all the French laboratories and to four other European laboratories. All the laboratories contacted agreed to participate (*n*=50). The results of the first test (May) were communicated to the laboratories (in July) before the second test was carried out (November). The French recommendations were available from the INCa web site (http://www.e-cancer.fr). Minor modifications to these recommendations were published between tests #1 and #2, and all the laboratories were informed of these changes.

All original molecular pathology reports were sent by post, fax and/or e-mail to Lincoln, for anonymisation. Anonymised reports were analysed by the organising laboratory and data the data were entered into the dedicated software. *BRAF* mutation status was entered twice, independently. Compliance with French recommendations was evaluated by analysing each original report and scoring 15 different parameters 0, 1, 2 or 3, corresponding to “absent”, “incomplete” and “complete”, respectively; Additional file 1: Table S1). Response time was determined as the interval between the day of delivery of the slide batches by the transporter and the day on which the corresponding report was received. The maximum acceptable response time was 40 days in test #1 and 28 days in test #2. A “good” response was defined as indicated in Table 1. This EQA did not evaluate the methods used by the various laboratories to assess *BRAF* status, but the collection of this information was optional.

**Table 1:** **Evaluation of the quality of responses for *BRAF *p.V600 mutational status**

**BRAF status**	**Response of the tested laboratory**	**Evaluation**
No p.V600 mutation	Absence of p.V600 mutation	Correct result
No p.V600 mutation	Presence of p.V600, p.V600E or p.V600K	Incorrect result
p.V600E	Presence of p.V600 or p.V600E	Correct result
p.V600E	Presence of p.V600K	Incorrect result
p.V600K	Presence of p.V600 or p.V600K	Correct result
p.V600K	Presence of p.V600E	Incorrect result

The quality of the DNA obtained from the FFPE samples was assessed after the completion of both tests. DNA was quantified by spectrophotometry (Nanodrop) and concentrations were adjusted to 25 ng/μl. Real-time PCR, generating an 80-base pair amplicon [15], was performed 14 times on each sample.

For the six samples of test #2, a deep sequencing analysis was performed, with the Ion AmpliSeq™ Cancer Hotspot Panel v2 primer pool and Ion AmpliSeq ™ Master Mix v2.0 (Ion Torrent, Life Technologies, Carlsbad, CA), according to the protocol recommended by the manufacturer. The multiplexed amplicon library concentration and size was determined with an Experion™ DNA analysis kit (Bio-Rad Laboratories Inc., Hercules, CA). Samples were barcoded with the Ion Xpress Barcode Adapters 1–16 Kit, according to the manufacturer’s instructions (Ion Torrent, Life Technologies) and multiplexed for emulsion PCR. Sequencing was performed with the Ion 316 Chip, on a Personal Genome Machine Sequencer (PGM, Ion Torrent, Life Technologies). The variants were characterised with the associated variant caller software.

Statistical analysis was performed with SAS software (SAS Institute, Cary, USA). Chi-squared or Fisher’s exact tests were used to assess differences for qualitative data, and analysis of variance or non-parametric Mann–Whitney tests were used to assess differences for quantitative data. All tests were two-tailed and a significance threshold of 5% was applied in all cases.

## Results

The 12 samples randomly selected for the tests corresponded to four cases with c.1799T>A, p.V600E, two cases with c.1798_1799GT>AA, p.V600K, and six cases without p.V600 *BRAF* mutations. The four European laboratories responded in due time in all cases and were evaluated only for *BRAF* status. The results were correct for 47/48, and false for one case (no mutation for a sample with a p.V600K mutation).

The following results concern only the 46 French laboratories, one of which participated only in the second test. We received 524 of the 546 responses expected within an acceptable timeframe (40 days for test #1, and 28 days for test #2). A technical failure of the determination of *BRAF* status was reported in four of these responses. Thus, overall, *BRAF* status was evaluated in an acceptable timeframe in 520 of 546 (95.2%) samples.

### BRAF mutation status

Correct results were obtained in 495 of these 520 responses (95.2%, 95% confidence interval [93.4-97.0]). Eleven of the false results were for p.V600, with confusion between p.V600E and p.V600K. This would have had no impact on treatment in Europe, where vemurafenib treatment is authorised for any p.V600 *BRAF* mutation. Fourteen of the 520 (2.7%, 95% confidence interval [1.3–4.1]) patients would have received incorrect results with a potential impact on treatment strategy. No false results were obtained for 25 of the 46 laboratories (one of which analysed only the six samples for the second test), 17 laboratories gave one false result, and four gave two false responses for the 12 samples tested. The correct result rate appeared to improve slightly between the first (249/263; 94.7%) and second (247/258; 95.7%) tests, but this different was not significant.

We matched the *BRAF* results with the position of the serial tissue sections, to check for possible tumour heterogeneity (Additional file 2: Figure S1). All sections for which false results were obtained were surrounded by sections for which good results were obtained, and the maximum thickness of tumours giving false results was 36 μm (3 batches of 3 slides, each 4 μm thick).

For the samples of test #2, one laboratory reported a minor (5%) c.1799T>A, p.V600E mutation in a wild-type sample, and additional *BRAF* c.1793C>T, p.Ala598Val, *BRAF* c.1807C>T, p.Arg603* mutations were reported by other laboratories. We checked these data by subjecting tumour DNA from the six samples to deep sequencing. The lowest sequence depth for the *BRAF* c.1793C to c.1807C region was 214 for the six samples (range [214 to 2246]), and the original mutational status of each sample was confirmed, excluding the possibility of additional and low-frequency mutations. All p.V600E mutations were also confirmed by immunohistochemistry with the VE1 antibody (not shown).

### Detection methods

Five laboratories changed their methods for *BRAF* p.V600 mutation detection between the two tests. Only one of these laboratories had had a false result in the first test. Fourteen different strategies were used, corresponding to combinations of one (52%), two (40%) or three (8%) of the following techniques: Sanger sequencing (37.9%), pyrosequencing (18.3%), high-resolution melting (HRM; 17.5%), allele-specific real-time PCR (15.3%), SNAPshot (9.5%) and Cobas (1.5%). All but one of these strategies included at least one technique developed in the laboratory concerned. None of the techniques used was associated with a significantly higher rate of false results (*P*=0.8; Additional file 1: Table S2).

### Correlation of false results with samples

**Figure 1:**
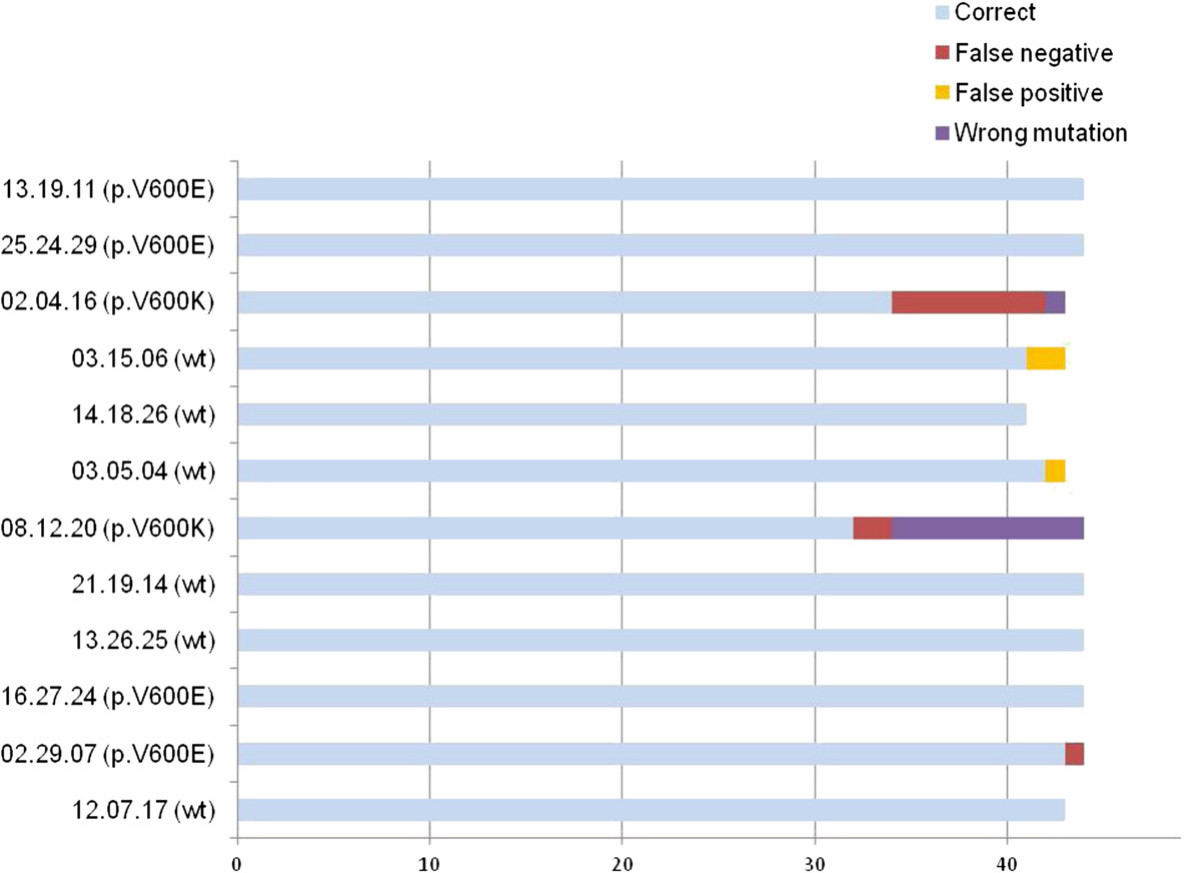
***BRAF *p.V600 status responses obtained from the 46 French laboratories.** Among the 12 samples tested, two had more than 20%, and three others had only a few (<5%) false responses.

The proportion of false results depended on the samples analysed and ranged from 0/44 (0%) for seven samples (3 with p.V600E and 4 with no mutation) to 12/44 (27.3%; Figure 1). The frequency of false results was highest for the two samples with p.V600K mutations (24.1% vs. 0.9%, *P*<0.001, Fisher’s exact test). We assessed the quality of the DNA obtained from FFPE samples, by comparing the number of DNA copies amplified by real-time PCR from each sample, at a given concentration of DNA. The mean CT values for the amplified DNA copies of the 12 samples were significantly different (*P*<0.0001, analysis of variance), ranging from 27.4 to 31.2. All but one of the false responses occurred in the six cases for which CT was highest (Additional file 1: Table S3), indicating low DNA quality.

### Response times - compliance with French recommendations

**Figure 2:**
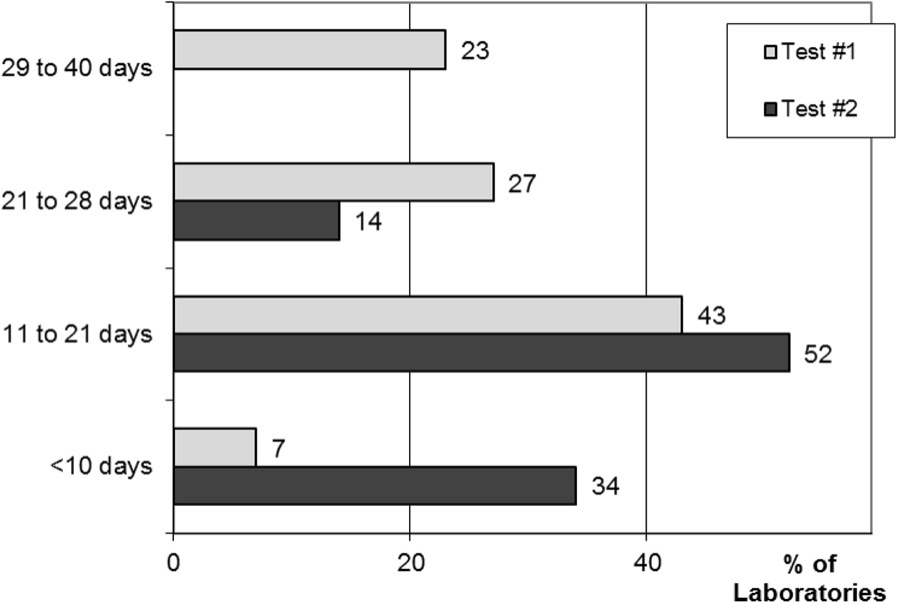
**Response time.** Response time, corresponding to the number of days between delivery by the transporter and reception of the report by the correspondent significantly improved (*P*<0.001) over the six-month period between the two tests (tests #1 and #2 were performed in May and November 2012, respectively).

Response times (Figure 2) improved between tests #1 and #2, from a mean of 22 to 12 days (*P*<0.001, Mann–Whitney test).

Compliance with French INCa recommendations was evaluated by calculating an overall score. The mean scores obtained were 33.2 and 33.5 for tests #1 and #2, respectively (maximum possible score = 40). No evaluation of the percentage of tumor cells was available for 60/263 (22.8%) and 8/260 (3.1%) of the samples in tests #1 and #2, respectively (*P*<0.001, Fisher’s test).

## Discussion

The health authorities of France (a country with 62 million inhabitants) have set up a network for the detection of somatic mutations in cancers, including *BRAF* p.V600 mutations in melanomas. This network carried out 3,479 tests for *BRAF* mutations in 2011 and 4,629 such tests in 2012. This study, the first EQA for this testing, revealed that only 2.7% of the results were false and had potential clinical implications, despite the use of techniques developed in the laboratory concerned and not certified, by most of the 46 laboratories. Most of the false results obtained related to two samples with a p.V600K mutation and poor DNA quality. Response time and pathological evaluations of samples improved significantly in the six months between the tests, as did the evaluation of tumour cell content.

The response time for tests of *BRAF* status is a major issue. Indeed, the median overall survival of patients with stage IV or advanced melanomas not treated with BRAF inhibitors is only six to 10 months [9]. Thus, treatment strategies, such as targeted or immune therapies, and/or possible inclusion in a clinical trial must be considered within the first month after diagnosis. This target in terms of timing is currently met by expert centres, but remains difficult to achieve in a nationwide network. The mean response time for test #1 was 22 days. This came as something of a surprise, because the expected response time was nine days, based on the data provided by each laboratory to INCa in 2011 (http://www.e-cancer.fr). The participating laboratories agreed to response decrease in the maximum response time from 40 to 28 days for test #2. In test #2, the mean response time was significant better, falling from 22 to 12 days (*P*<0.001). We attribute this important progress to the EQA and the discussion of the results obtained with all laboratories between tests #1 and #2. However, our findings cannot be considered to demonstrate a similar improvement of response times in routine practice. Quality management at all French medical laboratories, according to ISO 15189, and certification within the seven next years should also help to decrease response times.

This study revealed that almost all French laboratories are currently using tests developed in-house. Cost may be one of the chief reasons for this. Indeed, the use of a certified test to assess the *BRAF* status of tumour DNA costs at least twice as much as the use of in-house techniques [16]. However, the widespread use of non-certified detection methods might generate a high rate of false results. We show here that the false result rate was only 2.7%, only one third that for the pathological diagnosis of rare tumours in France (http://www.e-cancer.fr). Interestingly, the false result rate was not found to be related to the type of technique used, although the small number of samples (*n*=12) and the wide range of combinations of techniques used (*n*=17) limits our interpretation of this finding.

Twelve samples were tested, and 54%, 37% and 9% of the participating laboratories had no, one and two false results, respectively. Training at each laboratory is probably of considerable importance for limiting false results. Unfortunately, as the results were rendered anonymous for the EQA, we were unable to compare false result rates between laboratories as a function of their level of activity. The false result rate depended strongly on the sample and ranged from 0% to 27.3%. We therefore tried to identify parameters associated with a high false result rate. We found that the type of p.V600 mutation and the quality of the DNA obtained from the sample were significantly associated with the likelihood of false results. The false results rate was highest for the two cases with p.V600K mutations. These findings are consistent with those reported for another series, in which 30% of pV600K mutations were not detected by real-time PCR [17]. Real-time PCR with 25 ng/μl DNA revealed significant differences in the quality of the DNA obtained from the 12 samples (*P*<0.0001), with CT values from 27.4 to 31.2. The tissue must be fixed and embedded in paraffin for diagnosis in clinical practice, but these procedures may modify the nucleic acid [18]. Interestingly, false result rates of 1/260 and 24/261 were obtained for the six samples with the highest DNA quality and the six samples with the lowest DNA quality, respectively. There were too few samples to determine whether the type of p.V600 mutation and DNA quality had independent effects on the risk of a false result. However, our data suggest that the staff of these laboratories should be trained in the detection of rare p.V600 mutations, and consider routine testing the quality of DNA obtained from FFPE samples.

## Conclusions

In conclusion, we show here, for the first time, that nationwide EQA can improve the quality of molecular tests on FFPE tumour samples. We also show that, despite the use of several combinations of in-house tests, the false result rate for *BRAF* testing in melanoma was low. Finally, our data suggest that the training of laboratory staff to detect rare mutations and assessments of DNA quality might limit the risk of false results.

## Abbreviations

INCa: French National Institute for Cancer; EQA: External quality assessment; EGFR: Epidermal growth factor receptor; DNA: Eeoxyribonucleic acid; FFPE: Formalin-fixed paraffin-embedded; ISO: International Organisation for Standardisation.

## Competing interests

JFE, AL, PH, JCS and PLP received honoraria from Roche for expert advice on diagnosis and/or for studies on patients with melanomas. JFE received honoraria from Glaxo Smith Kline for expert advice on the diagnosis and/or treatment with BRAF inhibitors of patients with cancers. LB is employed by Roche.

## Authors’ contributions

JFE, JT and and LB designed the study, collected the data, contributed to data interpretation, wrote the manuscript. FN, SS, GF, AL, DL, ZHR, PH, JCS and PLP provided data, contributed to data interpretation. All authors read and approved the final manuscript. Members of the *BRAF* EQA Group provided data and contributed to data interpretation.

## Pre-publication history

The pre-publication history for this paper can be accessed here:

http://www.biomedcentral.com/1471-2407/13/472/prepub

## Supplementary Material

Additional file 1: Table S1 Evaluation of compliance with French recommendations. **Table S2**: False responses, by technique used 48% of the laboratories used a combination of two or three techniques to evaluate *BRAF* status. **Table S3**: *BRAF* p.V600 status and DNA quality of the FFPE melanoma samples. Click here for file

Additional file 2: Figure S1 *BRAF* p.V600 status results for serial tissue sections of the FFPE samples. In two cases (#08.12.20 and # 02.04.16) more than two false responses were obtained. Each false result was obtained for sections surrounded by sections that gave correct results, excluding the possibility of tumour heterogeneity. Click here for file
