# Supplementary material for: Improvement of the quality of BRAF testing in melanomas with nationwide external quality assessment, for the BRAF EQA group
Source: BMC Cancer. 2013 Oct 11;13:472. doi: 10.1186/1471-2407-13-472 (PMC3852250; doi:10.1186/1471-2407-13-472)
Supplement: Additional file 1: Table S1 — Evaluation of compliance with French recommendations. Table S2: False responses, by technique used 48% of the laboratories used a combination of two or three techniques to evaluate BRAF status. Table S3: BRAF p.V600 status and DNA quality of the FFPE melanoma samples. [file 1471-2407-13-472-S1.doc]

**Supplementary data**

Table s1: Evaluation of compliance with French recommendations

| Analysis of the report | SCORING | | |
| --- | --- | --- | --- |
| Item | Missing | incomplete | complete |
| Patient identification: first & last name, date of birth | 0 | 0 | 3 |
| Pathologist identification: first & last name, address | 0 | 2 | 3 |
| Sample Identification: alphanumeric digits | 0 | NA | 3 |
| Origin of sample: Primary tumour/metastasis | 0 | 1 | 1 |
| Type of sampling: surgery, biopsy, cytology | 0 | 1 | 1 |
| Histology: melanoma | 0 | 0 | 3 |
| Oncologist identification: first & last name, address | 0 | 1 | 2 |
| Type of analysis performed: *BRAF* V600 mutation | 0 | 1 | 3 |
| Percentage of tumour cells | 0 | NA | 3 |
| Unique internal number: alphanumeric digits | 0 | NA | 3 |
| Date sample received | 0 | 0 | 3 |
| Date of report | 0 | 0 | 3 |
| Unique internal number present on each page | 0 | NA | 3 |
| Detection method and its sensitivity | 0 | 1 | 3 |
| Conclusion | 0 | 1 | 3 |
|  |  |  |  |
|  |  | TOTAL | 40 |

**Table s2: False responses, by technique used**

48% of the laboratories used a combination of two or three techniques to evaluate *BRAF* status.

| Technique | Number | false |  |
| --- | --- | --- | --- |
| Sanger sequencing (in-house) | 311 | 14 | 4.5% |
| Pyrosequencing (in-house) | 150 | 5 | 3.3% |
| HRM (in-house) | 144 | 7 | 4.9% |
| Real-time PCR (in-house) | 126 | 6 | 4.8% |
| SnapShot (in-house) | 78 | 6 | 7.7% |
| Real-time PCR (Cobas) | 12 | 0 | 0.0% |

**Table s3: *BRAF* p.V600 status and DNA quality of the FFPE melanoma samples**

| Sample # | *BRAF*  status | Responses | | | RT01 | RT02 | RT03 | RT04 | RT05 | RT06 | RT07 | RT08 | RT09 | RT10 | RT11 | RT12 | RT13 | RT14 |
| --- | --- | --- | --- | --- | --- | --- | --- | --- | --- | --- | --- | --- | --- | --- | --- | --- | --- | --- |
|  |  | Correct | False | Total |  |  |  |  |  |  |  |  |  |  |  |  |  |  |
| 12.07.17 | wt | 43 | 0 | 43 | 28.3 | 28.2 | 28.4 | 28.5 | 28.4 | 28.3 | 28.4 | 28.3 | 28.4 | 28.4 | 28.4 | 28.4 | 28.9 | 28.9 |
| 02.29.07 | p.V600E | 43 | 1 | 44 | 31.0 | 31.0 | 30.8 | 30.9 | 31.0 | 31.5 | 31.2 | 30.9 | 31.0 | 31.4 | 31.5 | 31.2 | 31.6 | 31.7 |
| 16.27.24 | p.V600E | 44 | 0 | 44 | 29.5 | 29.7 | 29.8 | 29.7 | 29.5 | 29.8 | 29.4 | 29.3 | 29.9 | 29.9 | 30.0 | 29.8 | 30.2 | 30.1 |
| 13.26.25 | wt | 44 | 0 | 44 | 28.4 | 28.2 | 28.3 | 28.1 | 28.3 | 28.3 | 28.2 | 27.4 | 28.3 | 28.3 | 28.5 | 28.4 | 28.9 | 28.9 |
| 21.19.14 | wt | 44 | 0 | 44 | 27.0 | 27.0 | 27.4 | 27.6 | 27.4 | 27.3 | 27.4 | 27.4 | 27.4 | 27.3 | 27.4 | 27.3 | 27.7 | 27.8 |
| 08.12.20 | p.V600K | 32 | 12 | 44 | 29.1 | 29.2 | 29.1 | 29.4 | 29.4 | 29.6 | 29.1 | 29.2 | 29.5 | 29.6 | 29.5 | 29.6 | 30.0 | 29.9 |
| 03.05.04 | wt | 42 | 1 | 43 | 27.2 | 27.4 | 27.6 | 27.8 | 27.5 | 27.4 | 27.5 | 27.5 | 27.5 | 27.5 | 27.6 | 27.5 | 27.9 | 28.0 |
| 14.18.26 | wt | 41 | 0 | 41 | 28.0 | 27.9 | 28.0 | 28.0 | 28.1 | 28.0 | 27.9 | 27.9 | 28.5 | 28.2 | 28.2 | 28.2 | 28.7 | 28.7 |
| 03.15.06 | wt | 41 | 2 | 43 | 29.6 | 29.6 | 30.3 | 30.0 | 29.8 | 29.9 | 29.5 | 29.8 | 30.0 | 30.0 | 29.8 | 29.9 | 30.1 | 30.2 |
| 02.04.16 | p.V600K | 34 | 9 | 43 | 30.0 | 30.0 | 30.4 | 30.4 | 30.2 | 30.4 | 30.2 | 30.2 | 30.0 | 30.0 | 30.2 | 30.2 | 30.6 | 30.4 |
| 25.24.29 | p.V600E | 44 | 0 | 44 | 28.0 | 28.0 | 28.5 | 28.8 | 28.2 | 28.1 | 28.3 | 28.4 | 28.0 | 28.1 | 28.2 | 28.1 | 28.5 | 28.4 |
| 13.19.11 | p.V600E | 44 | 0 | 44 | 27.3 | 27.3 | 27.6 | 27.7 | 27.8 | 27.5 | 27.3 | 27.5 | 27.4 | 27.4 | 27.4 | 27.3 | 28.0 | 27.8 |
