# Supplementary figures and images for: Improvement of the quality of BRAF testing in melanomas with nationwide external quality assessment, for the BRAF EQA group
Source: BMC Cancer. 2013 Oct 11;13:472. doi: 10.1186/1471-2407-13-472 (PMC3852250; doi:10.1186/1471-2407-13-472)

## Slide 1
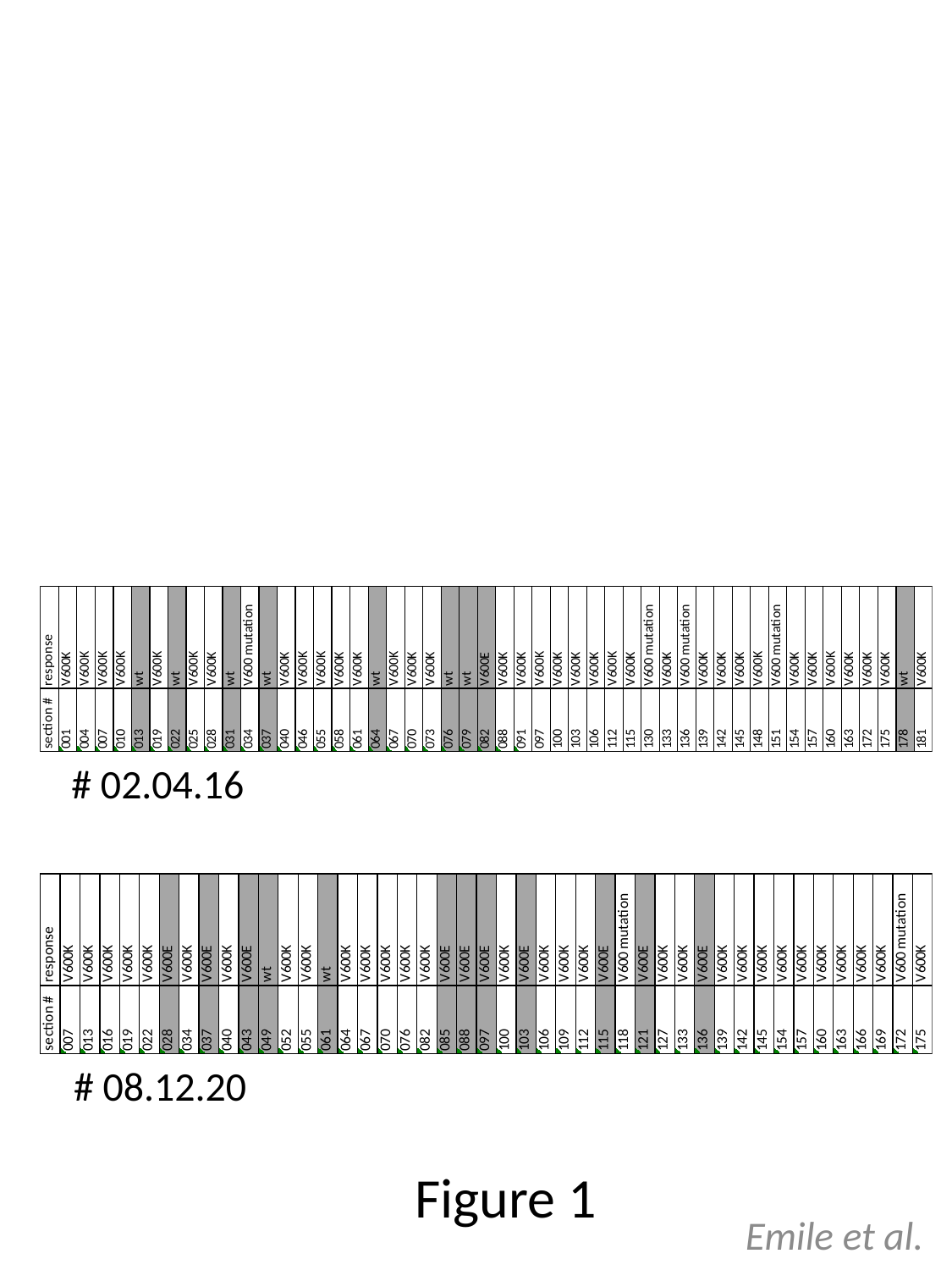

# 02.04.16
# 08.12.20
# Figure 1
Emile et al.

Supplement: Additional file 2: Figure S1 — BRAF p.V600 status results for serial tissue sections of the FFPE samples. In two cases (#08.12.20 and # 02.04.16) more than two false responses were obtained. Each false result was obtained for sections surrounded by sections that gave correct results, excluding the possibility of tumour heterogeneity. [file 1471-2407-13-472-S2.pptx]
